# Pathogenic variant of RBM20 in a multiplex family with hypertrophic cardiomyopathy

**DOI:** 10.1038/s41439-022-00183-z

**Published:** 2022-02-18

**Authors:** Natsuko Inagaki, Takeharu Hayashi, Yasuyoshi Takei, Hisanori Kosuge, Shinji Suzuki, Kousuke Tanimoto, Taishiro Chikamori, Akinori Kimura

**Affiliations:** 1 Department of Cardiology, Tokyo Medical University, Tokyo, Japan; 2 Department of Clinical Genetics Center, Tokyo Medical University, Tokyo, Japan; 3 Department of Physiology, Tokai University School of Medicine, Isehara, Japan; 4 Department of Molecular Pathogenesis, Medical Research Institute, Tokyo Medical and Dental University, Tokyo, Japan; 5 Department of Pediatrics and Adolescent Medicine, Tokyo Medical University, Tokyo, Japan; 6 Genome Laboratory, Medical Research Institute, Tokyo Medical and Dental University, Tokyo, Japan

**Keywords:** Cardiomyopathies, Medical genetics

## Abstract

*RBM20* is a disease-causing gene associated with dilated cardiomyopathy (DCM). The proband presented with the dilated phase of hypertrophic cardiomyopathy (HCM), and the mother also suffered from HCM. A missense variant of *RBM20*, p.Arg636His, previously reported as pathogenic in several families with DCM, was found in both the proband and the mother. Therefore, *RBM20* p.Arg636His could be the causative variant for this familial HCM, and *RBM20* might be a novel causative gene for HCM.

Hypertrophic cardiomyopathy (HCM) is characterized by unexplained left ventricular (LV) hypertrophy and diastolic dysfunction. On the other hand, dilated cardiomyopathy (DCM) is characterized by a dilated LV and decreased LV contractility. Most HCM patients show preserved LV contraction; however, some patients transition to a morphology similar to that of DCM with dilated ventricles, called the dilated phase of HCM (d-HCM)^1–3^. Furthermore, it is often difficult to differentiate between DCM and d-HCM owing to their similar morphologies. Moreover, the disease-causing genes of HCM, including those encoding sarcomeres, have also been registered as disease-causing genes of DCM, suggesting that there is some etiological overlap between HCM and DCM^4^.

A 41-year-old female proband patient (Fig. 1A, III-3) presented to our hospital with exertional dyspnea. We performed cardiac magnetic resonance (CMR) imaging using a Magnetom Skyra 3 T system (Siemens Healthineers, Erlangen, Germany)^5^ and found that the left ventricular end-diastolic diameter (LVDd) was dilated to 64 mm. In addition, the left ventricular end-systolic diameter (LVDs) was 48 mm, the diameter of the intraventricular septum (IVS) was 8.9 mm, the lateral wall diameter (LAD) was 6.5 mm, the left ventricular ejection fraction (LVEF) was reduced to 48%, and there was no coronary artery stenosis; therefore, we initially diagnosed the patient with DCM (Fig. 1B). In contrast, her 68-year-old mother (II-2, Fig. 1A) was asymptomatic and did not have hypertension, diabetes, or coronary stenosis. The mother’s CMR imaging revealed asymmetric septal thickening of the left ventricle, with a maximum IVS diameter of 13.2 mm and an LAD of 7.0 mm. The LVDd was 51 mm, and the LVEF was 54% (Fig. 1B). Consequently, we diagnosed the mother with HCM. With myocardial remodeling and deterioration due to fibrosis, the extracellular volume (ECV) increases, which can be quantitatively estimated by T1 mapping from CMR^6,7^. T1 mapping on a midventricular short-axis slice of myocardium was performed using the modified look-locker inversion recovery sequence before and after the intravenous administration of 0.1 mmol/kg gadobutrol (Gadovist, Bayer Healthcare, Leverkusen, Germany) as the MRI contrast agent, gadolinium. ECV was calculated using the following formula: ECV = (1-hematocrit) × (ΔR1_myocardium_/ΔR1_blood_), where R1 = 1/T1^6,7^. Hematocrit was measured before CMR imaging. These images were analyzed using Ziostation 2 (Ziosoft, Tokyo, Japan). The ECV fraction is usually higher when myocardial damage is more severe. ECV fraction mapping of the mother’s CMR image revealed an elevated ECV fraction, indicating myocardial remodeling and fibrosis centered in the IVS (Fig. 1B). The same procedure was performed on the proband’s CMR image, and the same ECV as that of her mother diagnosed with HCM was observed (Fig. 1B). Owing to the similarity of the increased ECV localization in both the mother and the daughter, we diagnosed the proband with d-HCM rather than DCM.

**Fig. 1:**
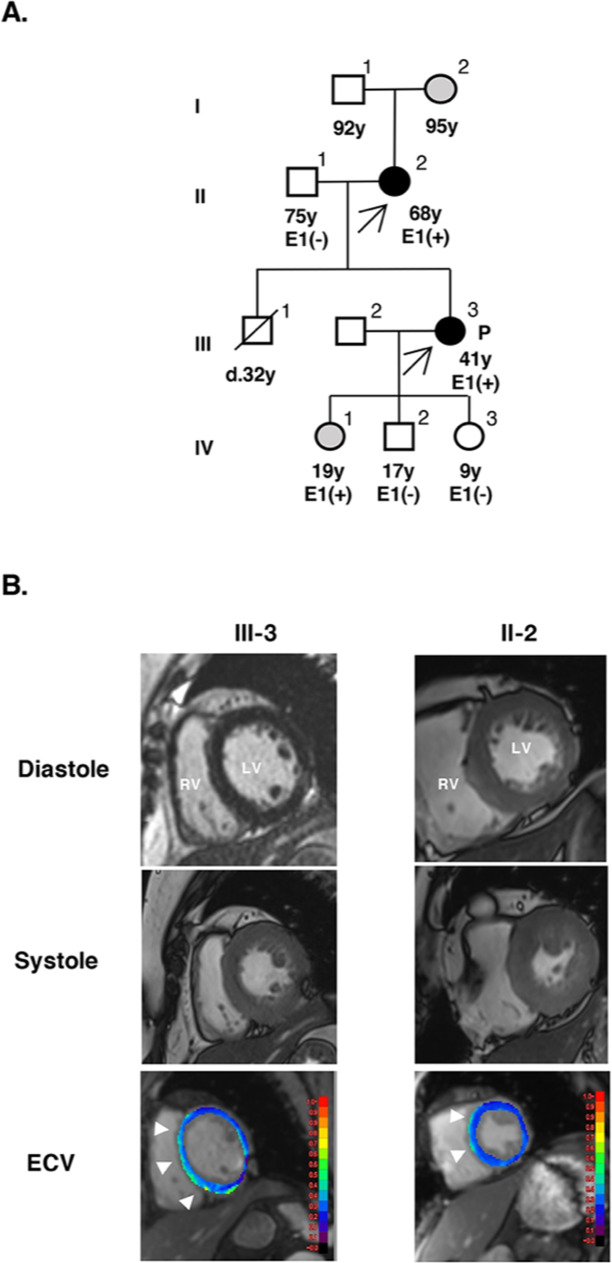
Clinical profile of a family with HCM. **A** Family pedigree showing the inheritance of cardiomyopathy. Squares denote males and circles denote females. Black symbols indicate affected individuals and open symbols indicate unaffected individuals. Gray symbols indicate individuals showing left ventricular hypertrophy on an electrocardiogram. The arrow indicates patients diagnosed with cardiomyopathy. P proband, d death, E genetic evaluation, + presence of *RBM20* variant, − absence of *RBM20* variant. **B** Short-axis images of cardiac magnetic resonance (CMR) for the proband (III-3) and her mother (II-2). The top and middle rows present end-diastolic and end-systolic cine images, respectively, and the bottom row shows extracellular volume (ECV) fraction mapping. Both III-3 and II-2 demonstrated elevated ECV (light green colored, arrowheads), primarily in the ventricular septum. RV right ventricle, LV left ventricle.

We conducted variant searches for 67 reported cardiomyopathy-causing genes in the peripheral blood genomic DNA of the proband patient who was diagnosed with d-HCM using an Ion Torrent PGM system (Thermo Fischer Scientific, CA, USA)^8^. This study was approved by the ethics committees of the local institutional review boards. A heterozygous missense variant, NM_001134363.3: c.1907 G > A, p.Arg636His, in the *RBM20* gene, which encodes the cardiac splicing factor RNA-binding motif protein 20, was identified and confirmed via Sanger sequencing (Fig. 2A). This variant was registered as rs267607004 in the dbSNP variant database. In the Genome Aggregation Database (gnomAD), which contains variants from the general population and multiple ethnic groups (https://gnomad.broadinstitute.org), the minor allele frequency of this variant is 0.000014, which is very rare; moreover, this variant is not registered in the Human Genetic Variation Database (HGVD), which is specific to the Japanese population (https://www.hgvd.genome.med.kyoto-u.ac.jp). This variant is located in an arginine/serine (RS)-rich region, specifically, the arginine-serine-arginine-serine-proline residues called the RSRSP stretch domain (Fig. 2B). The phosphorylation of the RSRSP stretch domain, which is a critical region, is required for the nuclear localization of *RBM20*^9^. Additionally, there have been previous reports of multiple families with DCM with the same variant, *RBM20* p.Arg636His, and the rare variant is registered as pathogenic/likely pathogenic in the ClinVar database (https://www.ncbi.nlm.nih.gov/clinvar/)^10,11^. Therefore, we concluded that *RBM20* p.Arg636His was the disease-causing variant in the proband. A family pedigree analysis of this variant revealed that the proband’s father (Fig. 1A, II-1), who had no heart disease, did not have the variant, whereas her mother (Fig. 1A, II-2), who was diagnosed with HCM, had the same variant. The proband’s daughter (Fig. 1A, IV-1) showed left ventricular hypertrophy on an electrocardiogram but normal echocardiography findings, and she had the same *RBM20* variant as her mother.

**Fig. 2:**
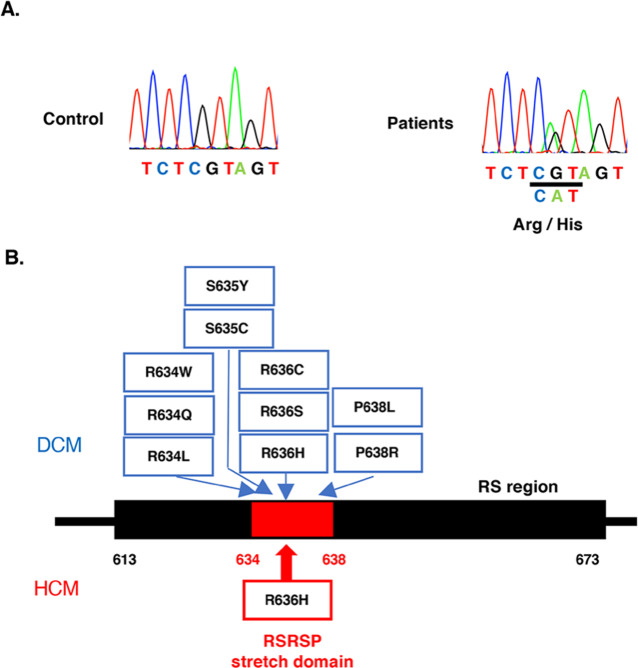
Identification of a pathogenic variant of *RBM20*. **A** Sanger sequencing results of the *RBM20* (NM_001134363.3) gene in healthy controls (left panel) and patients (right panel) are shown. The patients carry a heterozygous missense variant, NM_001134363.3 (RBM20_v001): c.1907 G > A, p.Arg636His. **B** In the RS region (marked in black) located in codons 613 to 673 of *RBM20*, all variants (upper row) of *RBM20* that were registered as “pathogenic” or “likely pathogenic” in the ClinVar database are located in the RSRSP stretch domain (codons 634 to 638), shown in red. These variants were all found in patients with DCM. The variant (*RBM20* p.R636H) found in this family with HCM (bottom row) is also located in the RSRSP stretch domain. DCM dilated cardiomyopathy, HCM hypertrophic cardiomyopathy.

*RBM20* is one of the major disease-causing genes in DCM^4,12^. The RBM20 protein is involved in splicing, particularly in regulating the splicing of *Titin*, which is a major causative gene of DCM^9^. Genetic variants of *RBM20* cause abnormal splicing of the *Titin* gene, which results in DCM. However, RBM20 is involved in the splicing of more than 30 genes, including sarcomere protein-coding genes such as *MYH7* and *TNNT2*, which are the major causative genes of HCM^9^. Additionally, some *RBM20* variants reportedly increase calcium sensitivity in the myocardium^13^. A number of HCM-causing variants in sarcomere genes increase calcium sensitivity in the myocardium^14,15^. These findings are consistent with the common functional alterations observed in HCM. Collectively, these results suggest that the *RBM20* p.Arg636His variant, which has been reported in families with DCM, could be the disease-causing variant in this family with HCM. The penetrance of the *RBM20* p.Arg636His variant has been reported to be 96–100% in patients over 30 years of age^10,11^. Therefore, the proband’s daughter (Fig. 1A, IV-1), carrying the same *RBM20* variant as the proband, has a high possibility of developing cardiomyopathy in the future and thus should be carefully monitored. In addition, it might be necessary to investigate whether the proband’s mother has any protective factor for myocardial dilatation. Moreover, subclinical cases of HCM in the reported families with DCM with the *RBM20* p.Arg636His variant may be identified in further family studies. Further studies are required, but the *RBM20* gene could be a potential cause of HCM.

## HGV database

The relevant data from this Data Report are hosted at the Human Genome Variation Database at 10.6084/m9.figshare.hgv.3122.
